# Influence of short-term face mask wear on semiautomatically measured tear film parameters in dry eye patients and healthy volunteers

**DOI:** 10.1007/s00417-022-05869-2

**Published:** 2022-10-21

**Authors:** Marc Schargus, E. M. Zimzik, L. Fuhrmann, G. Geerling

**Affiliations:** 1 Department of Ophthalmology, Heinrich-Heine University Düsseldorf, Moorenstraße, 5, 40225 Düsseldorf, Germany; 2 Department of Ophthalmology, Asklepios Hospital Nord-Heidberg, Hamburg, Germany

**Keywords:** Dry eye, NIBUT, Lipid layer thickness, Face masks

## Abstract

**Purpose:**

The use of face masks has been proposed to cause or exacerbate the symptoms of dry eye disease (DED), which has been widely discussed under the term mask-associated dry eye (MADE). However, no studies have systematically investigated tear film parameters during the use of different face masks. Therefore, the objective of the present study was to investigate clinically relevant parameters of the tear film before and during the short-time use of face masks in dry and normal eyes.

**Methods:**

In a prospective study, the tear film parameters of 42 DED patients and 42 healthy volunteers were examined while wearing different types of face masks in a randomized order. This included measurements of non-invasive tear break-up time (NIBUT), lipid layer thickness, tear meniscus height, and bulbar redness after 30 min of wearing no mask, a surgical face mask or an FFP2/K95 mask. The equivalence of the means was assessed using the two one-sided *t*-test (TOST) method.

**Results:**

In healthy volunteers’ lipid layer thickness, NIBUT and tear meniscus height were not significantly altered by 30 min of surgical or FFP2 mask wear (*p* > 0.016). The use of either type of mask was significantly associated with decreased bulbar redness (*p* < 0.001) in healthy eyes. In patients with DED, none of the tear film parameters or bulbar redness were significantly altered by 30 min of mask wear (*p* > 0.016).

**Conclusions:**

Based on these results, the short-term wearing of face masks, regardless of type, did not produce a significant difference in tear film parameters of lipid layer thickness, NIBUT, and tear meniscus in healthy or dry eyes, while bulbar redness was reduced after mask wear only in healthy volunteers.


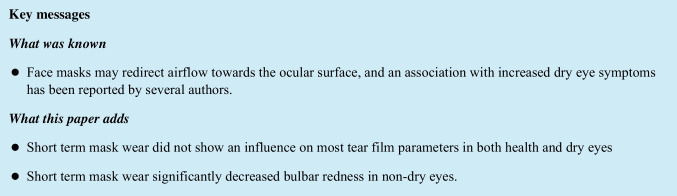


## Introduction

During the global COVID-19 pandemic, several publications have reported an increase in the incidence of dry eye disease (DED), and an association with the increased use of face masks and respirators has been considered [1–3]. The use of masks and respirators produced has been shown to result in reduced viral spread and risk of infection, and mandates for mask use are seen as an important public health intervention, especially in enclosed public spaces and public transport [4, 5].

During the first waves of the pandemic, the scarcity of certified personal protective equipment resulted in the use of a wide variety of materials and manufacturing techniques to produce masks for the general population. As the availability of three-ply nonwoven fabric surgical masks and FFP2/KN95 respirators has improved, these “medical” masks are widely used, and some European countries, such as Germany, specifically require their use in settings such as public transport or hospitals.

An association of the use of face masks with symptoms of dry eye has been reported. Recently, Krolo et al. reported significantly increased ocular surface disease index (OSDI) scores with a mask-wearing time of 3 h in non-dry eye patients [2]. Scalinici et al. demonstrated that patients with preexisting dry eye and long mask wear durations (> 6 h a day, 5 days a week) showed significantly higher OSDI scores compared to before the COVID-19 pandemic [3]. Boccardo assessed DED symptoms in 3605 face mask users by the use of a survey that was presented via social media platforms and showed that 18% of the participants reported increased symptoms of DED when wearing a face mask [6]. A proposed mechanism by which mask wear may affect the ocular surface is the redirection of expiratory airflow onto the ocular surface, exposing the tear film to mechanical disruption and increased evaporation and thus contributing to mask-associated dry eye (MADE) [7, 8]. Similar mechanisms have already been reported with the use of non-sealing continuous positive airway pressure (CPAP) masks in sleep apnea, although these produce greater airflow than medical face masks [9]. While several publications have investigated subjectively reported DED symptoms, the impact of face masks on objective tear film and ocular surface parameters in healthy and dry eyes has been limited.

## Materials and methods

### Participants, procedures, and parameters

Dry eye patients were consecutively recruited from the Dry Eye Clinic of University Eye Hospital Düsseldorf, Germany. Healthy study participants were recruited through public notices on the campus of the Heinrich-Heine University Düsseldorf, Germany. The general inclusion criteria were a minimum age of 18 years and informed consent, while exclusion criteria were a history of epilepsy, contact lens wear within 30 min before measurements, or ocular surgery within the last 4 weeks. The reason to define epilepsy as an exclusion criterion was that the flashing light of the LipiView® diagnostic device (TearScience Inc., Morrisville, NC, USA) was considered a possible trigger for photosensitive epileptic seizures. For the healthy group, inclusion criteria included a McMonnies questionnaire score of ≤ 14.5, with exclusion criteria of an established diagnosis of DED, systemic or ocular diseases directly relate to DED, use of any regularly applied eyedrops (e.g., treatment of glaucoma) or any other eyedrops with 2 h before the examination. The McMonnies dry eye questionnaire is a self-administered screening tool for dry eye disease which has been validated for use in the general population with good sensitivity and specificity [10]. For the DED group, inclusion criteria were a preexisting diagnosis of DED and a McMonnies score > 14.5, as this value has been validated as the threshold for positive DED screening in previous studies [10, 11].

After informed consent had been given in the written form, participants completed the McMonnies questionnaire, the OSDI questionnaire, and a medical history questionnaire, whereupon fulfillment of inclusion or exclusion criteria was checked. The medical history form recorded gender, age, preexisting ocular and systemic diseases, previous eye surgery, and use of spectacles. Spectacle wearers were instructed to continue to wear these throughout the entire data collection period.

Upon inclusion, the order of mask wear conditions (FFP2-mask/surgical mask/no mask) was randomized using sealed envelopes.

Surgical masks (Paul Hartmann AG, Heidenheim, Germany) consisted of a three-layer nonwoven fabric and a micro-germ filter complying with category II of the EU Regulation 2016/425 [12]. The FFP2 masks used (Paul Hartmann AG, Heidenheim, Germany) were 3-layer filtering respirators of non-woven fabric with a micro germ filter (category III of EU Regulation (EU) 2016/425) equivalent to N95 masks in the USA or KN95 in China [13] filtering at least 95% of airborne particles ≥ 0.3 µm size. Both mask types were fixed behind the ears with loop-shaped elastic bands and fitted to the nose using an integrated metal clip, and a tight fit was verified by the examiners.

Examinations were performed after 30 min of each mask wear condition as specified by the prespecified random order. Patients were asked to sit alone in a separate room during the mask-wearing period (Table 1). They were not allowed to use any electronic display use during this time to avoid blinking time changes. In the examinations without masks, the examiners wore tightly fitting FFP2 masks in agreement with local regulations. A separate shield to protect the investigator was not installed in order not to falsify the results.

**Table 1:** Possible mask wear and measurement sequences

Start	1. Wear test condition (30 min)	Measurements	2. Wear test condition (30 min)	Measurements	3. Wear test condition (30 min)	Measurements
Random assignment	No mask	→	Surgical mask	→	FFP2/N95 mask	→
	No mask	→	FFP2/N95 mask	→	Surgical mask	→
	Surgical mask	→	No mask	→	FFP2/N95 mask	→
	Surgical mask	→	FFP2/N95 mask	→	No mask	→
	FFP2/N95 mask	→	No mask	→	Surgical mask	→
	FFP2/N95 mask	→	Surgical mask	→	No mask	→

The following examinations were performed in a darkened room following the same protocol and sequence after 30 min in the morning between 8 and 12 AM. The right eye was always examined first; all examinations were done once per cycle. The lipid layer thickness was measured by interferometry (LipiView®, Johnson & Johnson Vision, Santa Ana, CA, USA) followed by an examination of non-invasive tear film break-up time (first breakup) (NIBUT), tear meniscus height, and bulbar redness using the Oculus Keratograph 5 M (Oculus Optikgeräte GmbH, Wetzlar, Germany).

All procedures performed met the ethical standards of the institutional research commission of the Heinrich-Heine University of Düsseldorf as well as the Helsinki Declaration of 1964 and its later amendments. The trial was registered with the DRKS (German Clinical Trials Register) (trial number: DRKS00024427) and approved by the Ethics Committee of the Heinrich-Heine University Düsseldorf on March 1st, 2021 (trial number: 2020–1219).

### Case number calculation

Case number calculations were performed using the R Core Team (2019, https://cran.r-project.org/), TOSTER package. NIBUT was chosen as a variable for sample size calculation and a difference in values of ≥ 2 s was determined to be clinically significant. A statistical significance threshold of 0.016 results in a required sample size of 42 subjects.

### Statistical analysis

Only data concerning the right eye of each participant was used for further evaluation. Statistical analysis was performed using IBM SPSS Statistics 25 (IBM Corp., Armonk, NY, USA), R Core Team, and StataCorp (Stata Statistical Software: Release 13. College Station, TX: StataCorp LP). For the comparison between healthy and DED groups under the condition of no mask wear, a significance level was set at *α* = 0.05. For the comparison of mask wear conditions, the significance level was set at *α* = 0.016 instead of the common *α* = 0.05 to allow multiple testing with three values (0.05/3 = 0.016) and to guard against spurious inference [14, 15]. This resulted in larger confidence intervals of 96.8% and 98.4%.

Agreement of metric variables was assessed by the use of Bland–Altman diagrams, which allow an evaluation of mean difference (MD) as well as the limits of agreement.[16]. Numerical, between and within variances limits of agreement, intraclass correlation [17], coefficient of repeatability, and coefficient of variation were calculated. A dependent (paired) *t*-test was used to assess statistical differences in mean measurements of different mask wear conditions. The equivalence of the mean values was evaluated using the TOST method (two one-sided tests) as established by Schuirmann in 1987 [18]. In this method, an upper (∆U = upper bound) and lower (∆L = lower bound) equivalence limit is defined as the smallest effect size of interest. On this basis, two null hypotheses (H01: ∆ ≤  − ∆L and H02: ∆ ≥ ∆U) are established. Two one-tailed *t*-tests were used to test whether the observed effect (∆) was within the defined equivalence limits. These were set at half the standard deviation of the differences, corresponding to a moderate effect with respect to Cohen’s d [19]. If the one-sided *t*-tests for the two null hypotheses can be rejected, it can be concluded that − ∆L < ∆ < ∆U holds. Thus, the observed effect (∆) was within the equivalence limits and therefore close enough to zero that two values can be considered statistically equivalent [20]. In the present case, this observed effect is the mean difference (MD) of two mask wear conditions.

## Results

Forty-two dry eye patients (32 female and 10 male) and 42 healthy volunteers (28 female and 14 male) were included in the study. The mean age was 52.4 in the DED group (range 24–81 years) and 24.8 years in the healthy group (range 19–46 years, *p* < 0.001, Mann–Whitney test) (Table 2). Spectacle use was reported by 12 (28.6%) of the healthy and 17 (40.5%) of DED participants, while contact lens use was reported by 7 (16.7%) of the healthy and 2 (4.8%) of DED participants.

**Table 2:** Average values and standard deviation (SD) of lipid layer thickness, non-invasive breakup time (NIBUT), tear meniscus height and bulbar redness of DED and healthy participants as measured after 30 min of each of the mask wear condition

	Healthy group	Dry eye group
Lipid layer thickness in nm (average, SD)		
FFP2 mask	67.33 (± 15.23)	78.34 (± 20.11)
Surgical mask	66.24 (± 17.4)	84.56 (± 16.58)
No mask	64.79 (± 18.56)	81.02 (± 19.27)
NIBUT in seconds (average, SD)		
FFP2 mask	16.01 (± 8.25)	9.9 (± 6.84)
Surgical mask	14.51 (± 7.81)	10.96 (± 9.67)
No mask	16.47 (± 7.96)	10.05 (± 6.56)
Tear meniscus height in mm (average, SD)		
FFP2 mask	0.29 (± 0.07)	0.38 (± 0.31)
Surgical mask	0.29 (± 0.07)	0.36 (± 0.23)
No mask	0.28 (± 0.76)	0.35 (± 0.2)
Bulbar redness in arbitrary units (average, SD)		
FFP2 mask	0.56 (± 0.22)	1.28 (± 0.51)
Surgical mask	0.56 (± 0.2)	1.38 (± 0.59)
No mask	0.66 (± 0.26)	1.34 (± 0.59)

The mean McMonnies score at baseline was 6.0 (SD 3.5, range 1–14) in the healthy group and 23.2 (SD 4.7, range 15–36) in the DED group (*p* < 0.01). The mean OSDI score was 8.0 (SD 7.6, range 0–33.3) in the healthy group and 40.1 (SD 21.8, range 0.0–81.8) in the DED group (*p* < 0.001, Mann–Whitney test).

### Lipid layer thickness

**Fig. 1:**
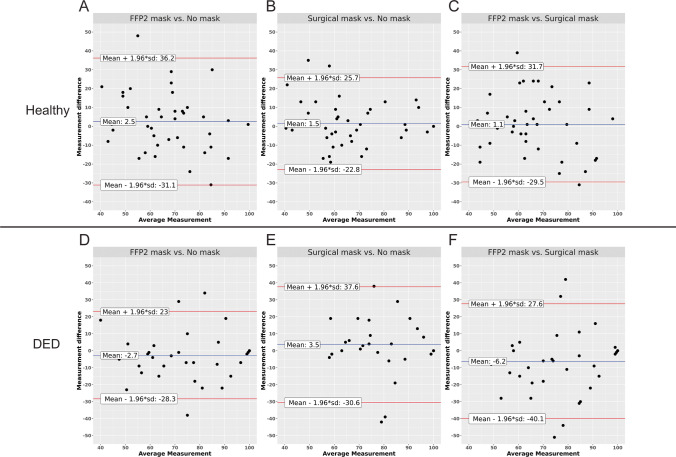
Bland–Altman diagrams of lipid layer thickness (LLT) measurements. Each dot represents the average of two LLT measurements (in nm, *x*-axis) of the same eye and the difference between them (*y*-axis). The horizontal blue line shows the mean difference of both measurement results, and the red lines show the limits of agreement. Panels **A–C** show measurements in healthy subjects, panels **D–F** show measurements of dry eye patients (DED). **A**, **D**: FFP2 mask vs. no mask; **B**, **E**: surgical mask vs. no mask; **C**, **F**: FFP2 mask vs. surgical mask

When no mask was worn, lipid layer thickness (LLT) was 64.79 nm in the healthy group and 81.02 in the DED group (*p* < 0.001). In the healthy group, the mean LLT during FFP2 mask use was 2.55 nm higher compared to no mask wear, a change which did not reach statistical significance and was deemed equivalent to zero upon TOST analysis (Fig. 1). The same applies to surgical mask wear compared to no mask wear (mean difference 1.45 nm, SD ± 12.38, *p* = 0.451) and FFP2 mask wear compared to surgical mask wear (mean difference 1.10 nm, SD ± 15.61, *p* = 0.652) (SD ± 17.18, *p* = 0.342) (Table 3).

**Table 3:** Mean difference of measurements, *p*-value of null hypothesis testing by paired *t*-test (NHST) (where *p* < 0.016 is defined as a statistically significant difference between the mask wear conditions), a *p*-value of two one-sided *t*-tests for statistical equivalence (TOST) (where *p* < 0.016 is defined as statistical equivalence between the mask wear conditions), and Pearson’s correlation coefficient for measurements of lipid layer thickness in nm, non-invasive tear break-up time (NIBUT) in seconds, tear meniscus height in millimeters, and bulbar redness in artificial units. *p*-values lower than the prespecified alpha of 0.016 are marked in bold

		Mean difference	NHST for statistical difference *p* of paired *t*-test	TOST for statistical equivalence *p* of less significant of two one-sided tests	Pearson’s correlation coefficient
Group	Lipid layer thickness (average) (nm)				
Healthy group	FFP2 mask no mask	2.56	0.342	**0.014**	0.50
	Surgical mask minus no mask	1.45	0.451	**0.008**	0.76
	FFP2 mask minus surgical mask	1.1	0.652	**0.004**	0.55
Dry eye group	FFP2 mask minus no mask	− 2.68	0.197	0.033	0.78
	Surgical mask minus no mask	3.54	0.200	0.032	0.54
	FFP2 mask minus surgical mask	− 6.22	0.026	0.188	0.57
	NIBUT (seconds)				
Healthy group	FFP2 mask minus no mask	− 0.46	0.710	**0.003**	0.52
	Surgical mask minus no mask	− 9.62	0.145	0.043	0.41
	FFP2 mask minus surgical mask	1.5	0.307	**0.016**	0.31
Dry eye group	FFP2 mask minus no mask	− 0.15	0.858	**0.002**	0.69
	Surgical mask minus no mask	0.91	0.533	**0.007**	0.40
	FFP2 mask minus surgical mask	− 1.06	0.512	**0.008**	0.27
	Tear meniscus height (mm)				
Healthy group	FFP2 mask minus no mask	0.04	0.719	**0.003**	0.63
	Surgical mask minus no mask	0.01	0.659	**0.004**	0.47
	FFP2 mask minus surgical mask	< − 0.01	0.881	**0.002**	0.6
Dry eye group	FFP2 mask minus no mask	0.03	0.480	**0.009**	0.45
	Surgical mask minus no mask	0.01	0.885	**0.002**	0.31
	FFP2 mask minus surgical mask	0.03	0.579	**0.006**	0.44
	Bulbar redness (arbitrary units)				
Healthy group	FFP2 mask minus no mask	− 0.1	** < 0.001**	0.865	0.81
	Surgical mask minus no mask	− 0.11	** < 0.001**	0.759	0.73
	FFP2 mask minus surgical mask	< − 0.01	0.834	**0.002**	0.77
Dry eye group	FFP2 mask minus no mask	− 0.10	0.264	0.023	0.81
	Surgical mask minus no mask	0.04	0.523	**0.007**	0.81
	FFP2 mask minus surgical mask	-0.10	0.153	0.044	0.71

**Fig. 2:**
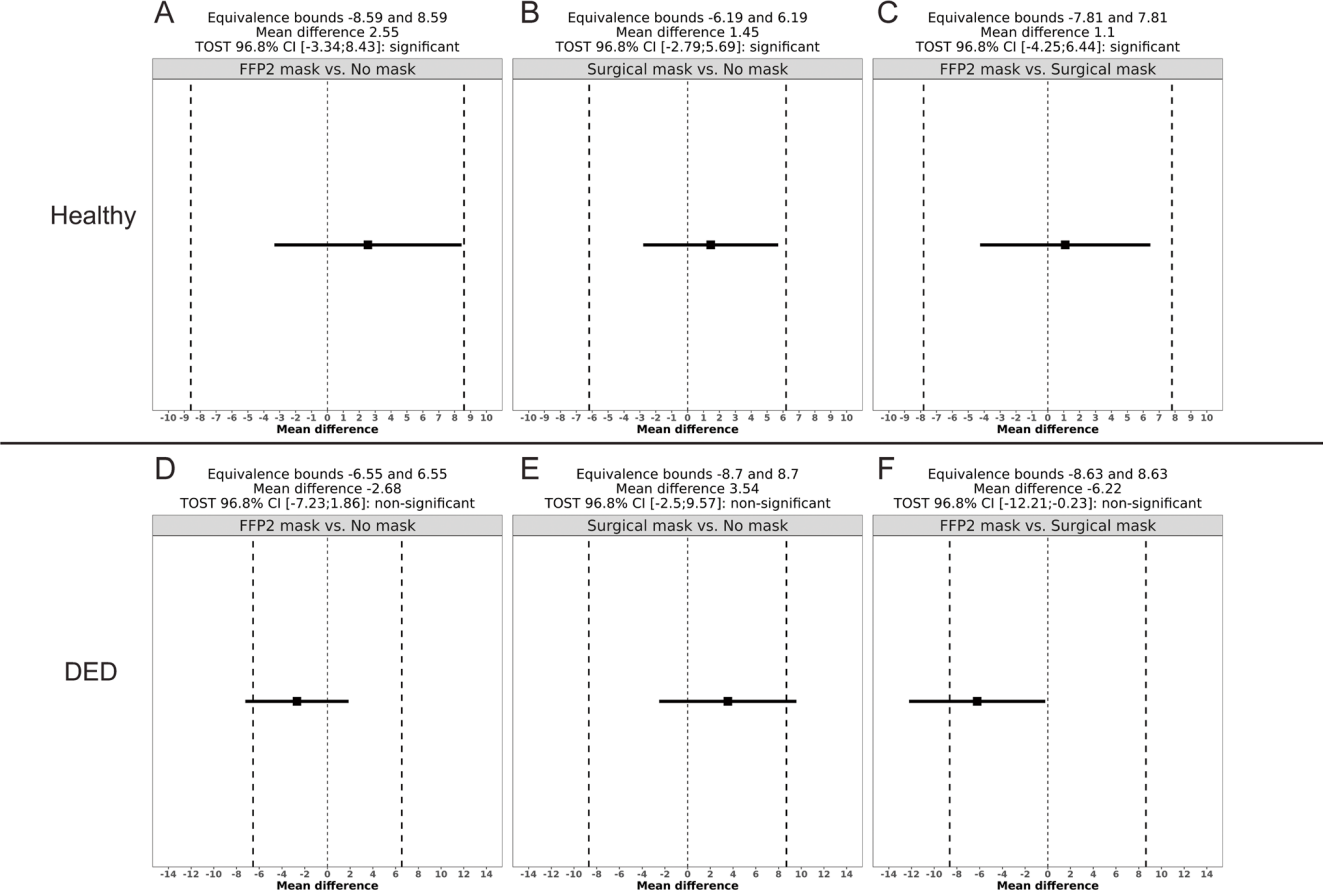
TOST of lipid layer thickness (LLT) measurements. The black square shows the mean difference of the two compared LLT measurements. The horizontal line shows the 96.8% confidence interval. The dashed vertical lines show the equivalence limits. Panels **A–C** show measurements in healthy subjects, panels **D–F** shows measurements of dry eye patients (DED). **A**, **D**: FFP2 mask vs. no mask; **B**, **E**: surgical mask vs. no mask; **C**, **F**: FFP2 mask vs. surgical mask

In the DED group, LLT also showed no significant differences in the three different test situations (Table 3). In TOST analysis, these comparisons were not deemed equivalent to zero as the TOST confidence intervals exceeded the equivalence bounds of plus or minus half a standard deviation from zero (*p* > 0.016) (Fig. 2). Therefore, while none of the comparisons revealed a significant change in LLT due to mask use, TOST analysis revealed the observed differences in healthy volunteers to be statistically equivalent to zero, while the possibility of a statistically non-equivalent difference which may be detectable in a greater number of participants could not be dismissed in DED patients.

### Non-invasive tear film break-up time

**Fig. 3:**
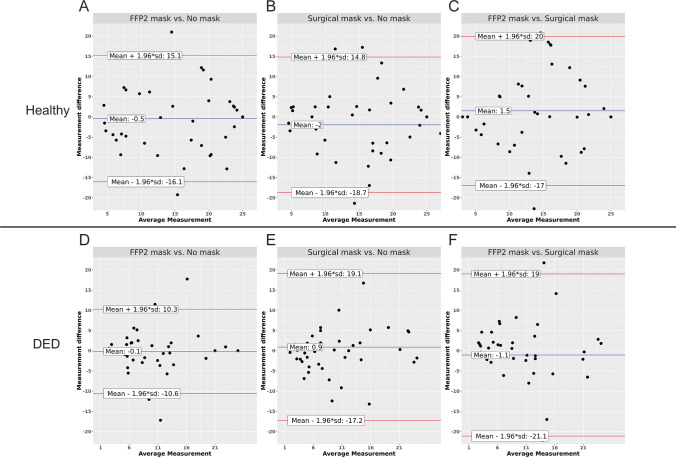
Bland–Altman diagrams of non-invasive tear break-up time measurements. Each dot represents an average of two NIBUT first break measurements (in seconds, *x*-axis) of the same eye and the difference between them (*y*-axis). The horizontal blue line shows the mean difference of both measurement results, and the red lines show the limits of agreement. Panels **A–C** show measurements in healthy subjects, panels **D–F** show measurements of dry eye patients (DED). **A**, **D**: FFP2 mask vs. no mask; **B**, **E**: surgical mask vs. no mask; **C**, **F**: FFP2 mask vs. surgical mask

**Fig. 4:**
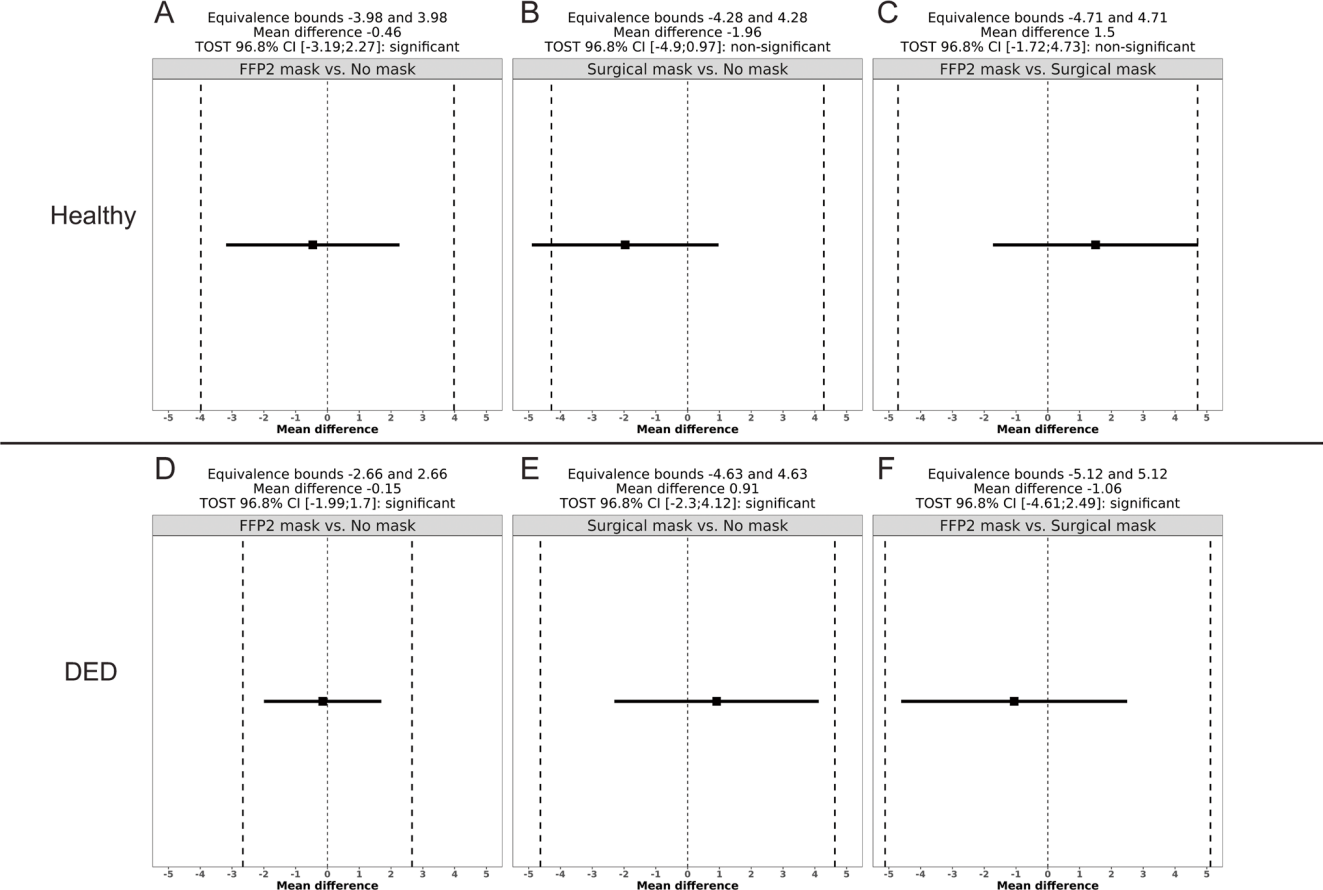
TOST of non-invasive tear break-up time measurements. The black square shows the mean difference of the two compared NIBUT measurements. The horizontal line shows the 96.8% confidence interval of the mean difference. The dashed vertical lines show the equivalence limits. Panels **A–C** show measurements in healthy subjects, panels **D-F** show measurements of dry eye patients (DED). **A**, **D**: FFP2 mask vs. no mask; **B**, **E**: surgical mask vs. no mask; **C**, **F**: FFP2 mask vs. surgical mask

At baseline, non-invasive tear film break-up time was significantly shorter in DED eyes at an average of 10.1 s compared to 16.5 s in healthy eyes (*p* < 0.001). None of the mask wear conditions resulted in a statistically significant change in NIBUT (all *p* > 0.016) (Table 3, Fig. 3). TOST analysis revealed the difference in TBUT between all mask wear conditions tested in DED patients to be equivalent to zero (*p* <  = 0.016). In healthy eyes, TOST analysis revealed only the comparison of FFP2 mask wear and no mask wear to be equivalent to zero (*p* <  = 0.016), while the hypothesis of non-equivalence could not be discarded for the other two comparisons between the mask wear conditions (*p* > 0.016) (Fig. 4).

### Tear meniscus height

Tear meniscus height was significantly higher in dry eyes (0.35 mm, SD ± 0.2) than in healthy eyes (0.28 mm, SD 0.08, *p* = 0.047) when no mask was worn. Tear meniscus height was not found to differ significantly depending on mask wear for any condition (*p* > 0.016). Upon TOST analysis, the tear meniscus height during all mask wear conditions, including no mask, was found to be statistically equivalent within the healthy and dry groups (Table 2).

### Bulbar redness

Bulbar redness was significantly greater in dry eyes (1.34, SD ± 0.59) compared to healthy eyes (0.66, SD 0.26, *p* < 0.001) when no mask was worn. In healthy eyes, bulbar redness was significantly reduced upon 30 min of wearing of either FFP2 masks (0.56, SD ± 0.22, *p* < 0.001) or surgical masks (0.56, SD ± 0.20, *p* < 0.001). These observed differences were statistically not equivalent to zero upon TOST analysis (FFP2 mask compared to no mask: *p* = 0.865, surgical mask compared to no mask: *p* = 0.759). In dry eyes, bulbar redness was not significantly different depending on the different types of masks worn (*p* > 0.016).

As shown in Table 3, all pairwise comparisons of lipid layer thickness, NIBUT, and tear meniscus height between the mask wear conditions were not statistically different (*p* > 0.016) in either group, while the results of the equivalence testing by TOST varied. Comparisons by null-hypothesis significance testing (NHST) were statistically significant (*p* > 0.016) only for the bulbar redness in healthy participants.

## Discussion

This study compared the effect of no mask wear to surgical and FFP2 mask wear of 30-min duration on lipid layer thickness, NIBUT, tear meniscus height, and bulbar redness in a healthy young cohort and a cohort of DED patients using the LipiView® interferometer and Keratograph 5 M. Previous on this topic have largely focused on symptom-oriented questionnaires, and few objective tear film examination methods have been performed [2, 3, 6]. The effect of different mask types (surgical masks, FFP2/(K)N95) had also not been studied so far.

Our results have shown most tear film parameters to be unaffected by short-term use of either mask type. Notably, only bulbar redness was shown to be significantly reduced in healthy adults upon mask wear. This was surprising, as MADE is generally thought to be associated with ocular surface irritation, which would be expected to result in increased bulbar redness as a consequence of conjunctival vasodilatation. A possible explanation may be that exposure to increased airflow and evaporation affects the ocular surface temperature which may trigger a vasoconstriction of conjunctival vessels and hence increase the visually detectable redness of the eyes. No significant change in bulbar redness was revealed in the dry eye group, which may be due to their greater degree of bulbar redness at baseline, as the presence of DED as a trigger of bulbar redness may have prevented a significant decrease due to mask wear. Certainly, it seems prudent to recognize the possibility of mask wear playing a confounding role in studies which include measures of bulbar redness, and future studies may explore the role of ocular surface temperature associated with face mask use.

Previous studies have investigated the effects of face mask wear on the ocular surface by several different methodologies. Boccardo et al. reported an incidence of new MADE symptoms in face mask wearers of 18% [6]. An Israeli study using thermal video recordings confirmed that face masks direct the airflow from their upper edge toward the eye [21]. Patel et al. were able to show that sealing the upper edge of the mask using an adhesive tape reduces the number of colony-forming units directed toward culture plates mounted above the eyes [22]. Others have reported improvement in dry eye symptoms with the use of face masks and shields, which could be explained by the increase in the moisture level around the eye by breathing air [23]. Mastropasqua et al. reported a reduced tear break-up time, increased ocular surface staining (fluorescein and lissamine), and inflammatory markers (dendritic cells in confocal microscopy and HLA-DR expression in impression cytology) after 3 months in patients wearing face masks for more than 3 h daily, especially in patients with preexisting dry eye. In healthy individuals, this was detectable to a lesser extent and only with extensive mask wear (beyond 6 h) [24]. However, masks are frequently worn for a shorter period only to prevent direct viral transmission to others.

Arriola-Villalobos et al. reported an increase in NIBUT measured by the Keratograph 5 M in patients with DED following the removal of previously worn face masks [25]. This finding was not observed in our study after 30-min wear of either mask type. It should be noted that the previous study did not report the duration or type of mask worn prior to NIBUT measurement, and the sequence of mask wear or no mask wear was not randomized. Additionally, there was no control group in which masks were not removed. The observed increase in NIBUT after mask removal may therefore have occurred after prolonged use of the masks or due to a temporal coincidence with an improved NIBUT after the introduction of the participants to the controlled study environment.

A limiting factor in our study was that the masks were worn for 30 min only prior to measurements. This short duration was chosen due to the fact that short-term mask wear in specific environments, such as public transport or health facilities, is widespread in the general population, as well as being relevant to a large proportion of participants in other clinical studies in which tear film analysis is employed. Consequently, the results cannot be generalized toward other patterns of mask use, and the indoor location of the measurement means that they cannot be generalized to other environments, especially outdoors. At baseline, a greater mean tear meniscus height and mean LLT was noticed in the DED group, which may be due to reactive tear production and the inclusion of patients undergoing treatment with lubricating eyedrops, although these were not applied in the 2 h before testing. In addition, it should be mentioned that people wearing spectacles have been included in this study. Although they were asked to wear their glasses between the examinations, it cannot be excluded that wearing spectacles may represent a confounder for these measurements. While attention was paid to a tight fit of the masks by the investigators, it is known that the fit of the masks underlies some variation over time and mask fit in real life is not usually checked by a second person as it was in this study setting. The possible effects of suboptimal mask fit may thus be even more pronounced in real life than that in our study. Furthermore, the differences in age and gender between the healthy and dry eye groups in our study limit the comparability between the groups. We deliberately chose to compare a cohort of generally older dry eye patients to a younger healthy cohort in order to maximize the hypothesized vulnerability toward dry-eye-related alterations of tear film parameters, but due to this confounding effects of the differences in group composition are possible. It should also be noted that semiautomatic non-invasive examination of the ocular surface is not yet a substitute for clinical examination methods. Beside several studies showing a good correlation between the OSDI and non-invasive tear film tests, there are also examples of multicenter studies with Keratograph examinations that did not show any correlation [26–28].

Overall, our study has shown that short-term mask use is not associated with changes in most clinically relevant tear film parameters. While conclusions concerning long-term use cannot be drawn from these results, they are relevant to the context of clinical studies. It was previously unclear whether the use of face masks may impose an influence on tear film parameter measurements in studies that may or may not be related to DED, and the use and type of masks have been inconsistently reported in such settings. As demonstrated by the statistical equivalence of the reported tear film, the use of masks does not appear to be a confounding factor in automated tear film analysis, although this does not apply to measurements of bulbar redness where a surprising reduction associated with mask wear was observed.
